# Bioinformatics analysis of potential key genes and pathways in neonatal necrotizing enterocolitis

**DOI:** 10.1186/s12887-022-03721-4

**Published:** 2022-11-12

**Authors:** Xuexiu Liu, Xianhong Zhang, Luquan Li, Jianhui Wang, Yanhan Chen, Liping Wu

**Affiliations:** 1 Department of Neonatal Diagnosis and Treatment Center, Children’s Hospital of Chongqing Medical University, National Clinical Research Center for Child Health and Disorders, Ministry of Education Key Laboratory of Child Development and Disorders; China International Science and Technology Cooperation Base of Child Development and Critical Disorders; Chongqing Key Laboratory of Pediatrics, Chongqing, 400014 P.R. China; 2 College of Nursing, Chongqing Medical University, Chongqing, 400016 P.R. China; 3 Department of Nursing, Children’s Hospital of Chongqing Medical University, National Clinical Research Center for Child Health and Disorders, Ministry of Education Key Laboratory of Child Development and Disorders; China International Science and Technology Cooperation Base of Child Development and Critical Disorders; Chongqing Key Laboratory of Pediatrics, Chongqing, 400014 P.R. China

**Keywords:** Neonatal necrotizing enterocolitis, Bioinformatics analysis, Differentially expressed genes, Gene chip

## Abstract

**Objective:**

To detect differentially expressed genes in patients with neonatal necrotizing enterocolitis (NEC) by bioinformatics methods and to provide new ideas and research directions for the prevention, early diagnosis and treatment of NEC.

**Methods:**

Gene chip data were downloaded from the Gene Expression Omnibus database. The genes that were differentially expressed in NEC compared with normal intestinal tissues were screened with GEO2R. The functions, pathway enrichment and protein interactions of these genes were analyzed with DAVID and STRING. Then, the core network genes and significant protein interaction modules were detected using Cytoscape software.

**Results:**

Overall, a total of 236 differentially expressed genes were detected, including 225 upregulated genes and 11 downregulated genes, and GO and KEGG enrichment analyses were performed. The results indicated that the upregulated differentially expressed genes were related to the dimerization activity of proteins, while the downregulated differentially expressed genes were related to the activity of cholesterol transporters. KEGG enrichment analysis revealed that the differentially expressed genes were significantly concentrated in metabolism, fat digestion and absorption pathways. Through STRING analysis, 9 key genes in the protein network interaction map were identified: EPCAM, CDH1, CFTR, IL-6, APOB, APOC3, APOA4, SLC2A and NR1H4.

**Conclusion:**

Metabolic pathways and biological processes may play important roles in the development of NEC. The screening of possible core targets by bioinformatics is helpful in clarifying the pathogenesis of NEC at the gene level and in providing references for further research.

Neonatal necrotizing enterocolitis (NEC) is caused by ischemia and hypoxia of the intestinal wall and results from a variety of factors that damage the blood supply of the intestinal mucosa. NEC mainly impacts preterm infants, especially those with very low birth weight. The incidence rate ranges from 7 to 11% and is associated with high mortality [1]. It has been reported that the mortality of NEC children with low birth weight was 41.7%, while that of children with very low birth weight was 50.2% [2]. Furthermore, the mortality of children with extensive intestinal necrosis is nearly 100%, which is a significant cause of neonatal death [3]. After years of effort, the mortality rate remains at 20% to 30% and is even higher in cases requiring surgical treatment [4]. NEC can damage children’s intellectual development and is an important risk factor for long-term intellectual disability [5, 6]. With regard to diagnosis and treatment, it is often difficult to accurately predict intestinal necrosis in the early stage of the disease by using a single indicator, while the combined application of multiple indicators helps more [7, 8]. Bell et al. formulated the NEC diagnostic standard in 1978 [9], and Walsh and Kliegman revised it in 1987 [10]. However, in view of the fact that NEC has had many manifestations in recent years [11], both the "two out of three" standard for premature infants developed by the International Association of Newborns and the Vermont Oxford Network diagnostic standard based on Bell staging have included abdominal B-ultrasound examination [12]. However, these are far from sufficient for the early diagnosis of NEC in clinical practice. The pathogenesis of NEC has been documented to be closely related to genetic factors [13]. Recent developments in molecular analysis techniques have increased the availability of more advanced genetic analysis methods in laboratory or even clinical circumstances, making a more comprehensive and accurate diagnosis of NEC possible at its earlier stages. However, to the best of our knowledge, the literature on this topic is still limited.

To establish a hybrid gene analysis method to early reveal NEC, this study, based on the Gene Expression Omnibus (GEO) and using the GSE46619 dataset, aimed to identify the differentially expressed genes (DEGs) in children with NEC by bioinformatics methods. Functional (GO), signal pathway (KEGG) and protein‒protein interaction (PPI) network integration analyses of differentially expressed genes were also performed, and meaningful PPI modules and key genes were detected. This study provides a reference for further research on the molecular mechanism of NEC.

## Material and methods

### Data sources

The GeneChip dataset GSE46619 was downloaded from the GEO database (http://www.ncbi.nlm.nih.gov/geo/), which was submitted by NGPC, CHAN KY, LEUNG KT, et al., and included 9 samples from patients, 5 of whom underwent surgery on intestinal tissues and 4 control cases consisting of patients with other noninflammatory congenital intestinal diseases who underwent other surgeries.

### DEG screening

The GSE46619 dataset was analyzed with the online network analysis tool GEOR2 in the GEO database. DEGs with adjusted *P* < 0.05 and |log_2_ FC|> 2 were selected.

### Bioinformatics analysis of DEGs

GO enrichment and KEGG pathway analyses were performed on the related DEGs in NEC using the online analysis software DAVID (https://david.ncifcrf.gov/). The differences were statistically significant when *P* < 0.05.

### Construction and analysis of the PPI network

A PPI network was used to facilitate the analysis of gene or protein interactions, while the STRING online database (http://stringdb.org/) was used to help construct the PPI network diagram. To reveal the potential interactions between differentially expressed genes, the PPI network diagram data constructed by STRING were imported into Cytoscape software, while the mcode plug-in was used to analyze the subnetwork structure of the PPIs. Degree of connectivity is a parameter used to evaluate the connection tightness in a network. The higher the connectivity is, the closer the connection and the stronger the interaction with other nodes in the whole network, thus enhancing the stability of the whole network. Those genes with higher connectivity will be regarded as the key ones among the differentially expressed genes. The three most significant modules and key genes were screened out using the MCODE plug-in of Cytoscape.

## Results

### DEG screening

Based on *P* < 0.05 and |log2FCΙ|> 2, 236 differentially expressed genes were screened, including 225 upregulated genes and 11 downregulated genes. The top 10 differentially expressed genes are shown in Table 1.

**Table 1:** Up regulation of DEG(TOP10)

Serial number	*P* value	Gene name	Gene annotation
1	0.0000713	CLRN3	clarin3
2	0.00007123	MTTP	microsomal triglyceride transfer protein
3	0.0000711	ALDOB	aldolase, fructose-bisphosphate B
4	0.00007093	ANXA13	annexin A13
5	0.00007093	OTC	ornithine carbamoyltransferase
6	0.00007093	APOA4	apolipoprotein A4
7	0.00006994	FABP2	fatty acid binding protein 2
8	0.00006994	PRSS8	protease, serine 8
9	0.00006958	AADAC	arylacetamide deacetylase
10	0.0000623	VIL1	Villin 1

### GO enrichment and KEGG analyses of DEGs

**Fig. 1:**
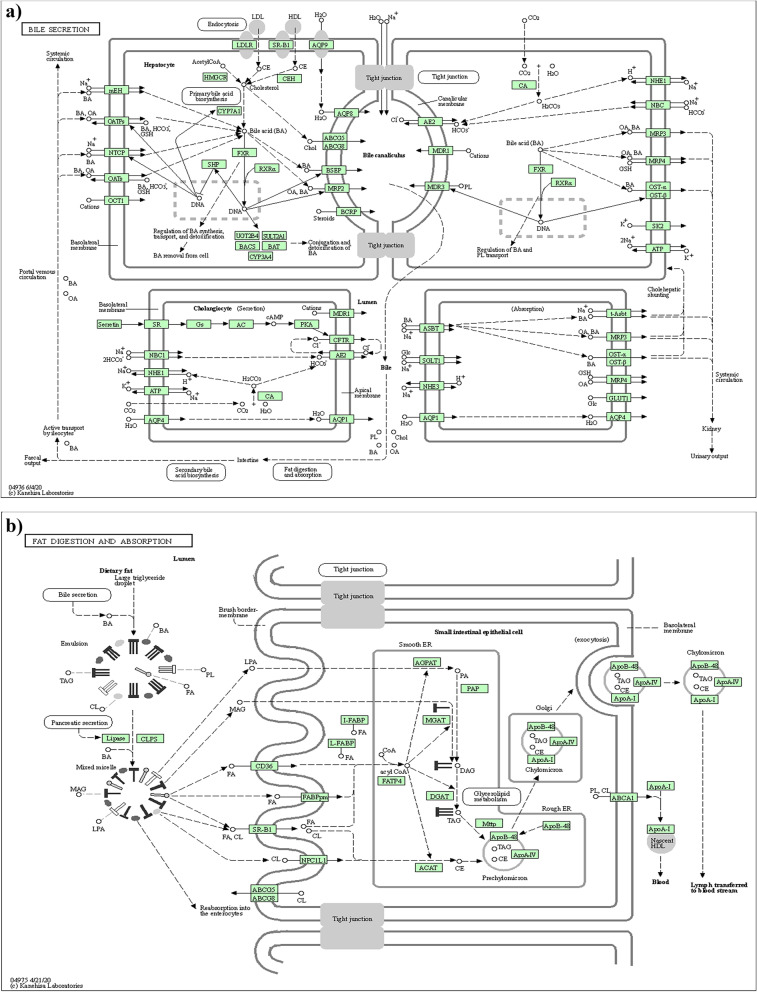
Schematic Diagram of the KEGG Pathways (**a**, **b**)

GO enrichment and KEGG analyses were performed on 236 DEGs with the DAVID online tool, and the results are displayed in Tables 2 and 3. (1) Biological process: Differentially expressed genes were significantly concentrated in the processes of heterologous metabolism, drug response, redox process, inflammatory reaction, and carbohydrate metabolism, among others. (2) Cell component: Differentially expressed genes were dramatically accumulated in the membrane, exosome, plasma membrane, plasma membrane, and sarcoplasmic membrane components, among others. (3) Molecular function: Differentially expressed genes were significantly concentrated in protein homodimeric activity, transporting activity, actin filament binding, iron ion binding, actin binding, and monooxygenase activity, among others. KEGG enrichment analysis demonstrated that the differentially expressed genes were significantly enriched in the metabolism, fat digestion and absorption, protein digestion and absorption, chemical carcinogenesis, carbohydrate digestion and absorption, and retinol metabolism pathways (Fig. 1).

**Table 2:** KEGG analysis of DEGs

Pathway	Description	Gene number	P	Gene
hsa04975	Fat digestion and absorption	9	2.61e-07	ABCG8. FABP2. ABCG5. DGAT1. MOGAT3. MOGAT2. MTTP. APOA4. APOB
hsa04976	Bile secretion	9	1.19e-0.5	ABCG8. S4C9A3. ABCG5. ABCB1. NRIH4. ATPIA1. S4C51B. CFTR. SULT2A1
hsa01100	Metabolic pathways	35	1.19e-0.5	SDS1. ST6GALNAC1. DDC. TPH1. ACY1. MOGAT3. GDA. GBA3. CYP2C19. FUT2. PTGS2
hsa04974	Protein digestion and absorption	8	0.00039	S6C9A3. ACE2. S4CI5A1. S4C6A19. MEPIA. KCVJ13. S4CIA1. ATPIA1
Hsa04973	Carbohydrate digestion and absorption	6	0.00039	HKDC1. S1. S4C2A2. ATPIA1. 4CT. SLIC37A4

**Table 3:** GO analysis of DEGs

Type	Classification number	Gene numbe	*P* value	Gene function
GOTERM—BP	GO: 0,007,586	13	8.25e-07	Digestion
	GO: 0,015,711	22	1.58e-06	Organic anion transport
	GO:0,006,629	38	1.58e-0.6	Lipid metabolism process
	GO:0,044,281	47	2.19e-0.6	Metabolic process of small molecules
	GO:0,006,820	24	2.19e-0.6	Anion transport
GOTERM – BMF	GO:0,008,509	17	0.00011	Activity of anion transmembrane transporters
	GO:0,005,215	33	0.00021	Transport activities
	GO:0,022,804	16	0.00034	Active transmembrane transporter activity
	GO:0,022,857	29	0.00035	Transmembrane transporter activity
	GO:0,051,015	10	0.0013	Actin filament binding
GOTERM—CC	GO:0,045,177	32	7.61e-17	The apical part of a cell
	GO:0,016,324	29	2.38e-10	Parietal plasma membrane
	GO:0,005,903	15	7.50e-11	Brush border
	GO:0,098,590	40	2.22e-10	Plasma membrane area
	GO:0,044,425	113	6.96e-09	Membranous part

### PPI analysis

**Fig. 2:**
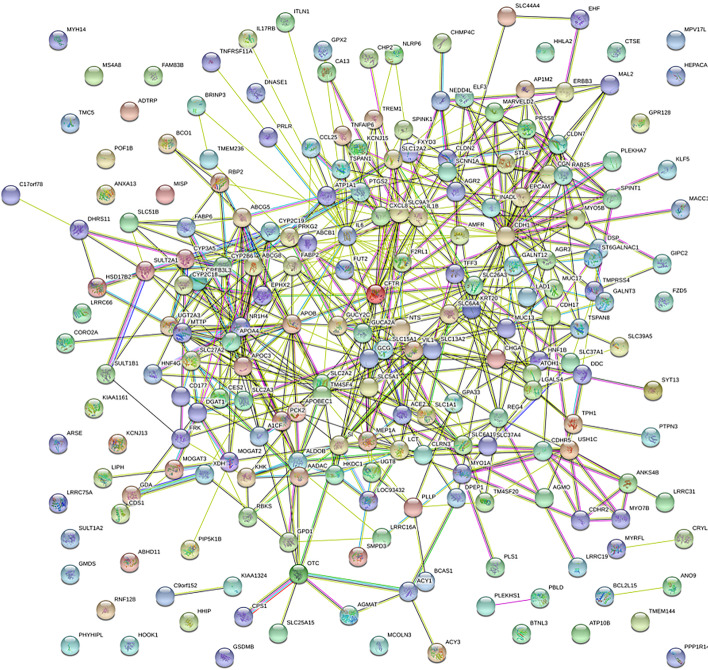
Schematic Diagram of the PPI Network

**Fig. 3:**
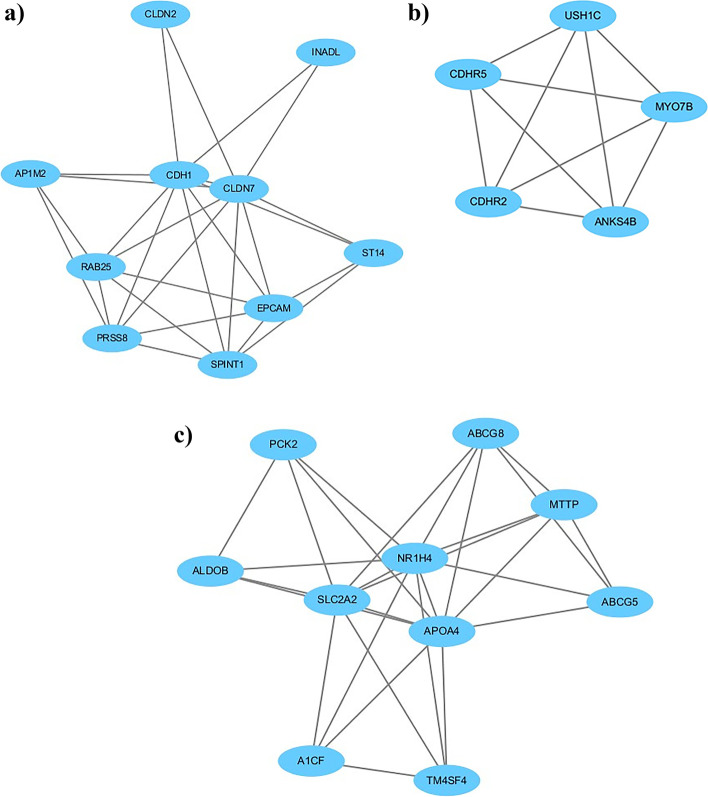
Schematic Diagram of the Modules (**a**,**b**,**c**)

All differentially expressed genes were uploaded to the STRING database, and PPI network data were obtained. The PPI network data were imported into Cytoscape software for further analysis of the potential biological information of the PPI network diagram, and the key factors of the nine hub nodes with the highest value > 15 were selected, which included epithelial cell adhesion molecule (EPCAM), cadherin 1 (CDH1), cystic fibrosis transmembrane conduction regulator (CFTR), interleukin-6 (IL-6), APOB, APOC_3_, apolipoprotein A-IV (APOA_4_), solute carrier family 2-facilitated glucose transporter member 1 (SLC2A), and nuclear receptor subfamily 1, group H, member 4 (NR1H_4_). Two genes were downregulated, and seven genes were upregulated. A PPI network was constructed (Fig. 2) with 204 nodal proteins and 528 edges, and *p* < 0.001. The PPI network diagram was analyzed using the MCODE plug-in of Cytoscape, and the three most significant modules were selected (Fig. 3).

## Discussion

In this study, bioinformatics analysis was performed on the gene expression profiles of intestinal tissues of 5 NEC patients and 4 non-NEC patients, and the differentially expressed genes involved in the occurrence and development of NEC were identified. Compared with those of the control group, 225 differentially expressed genes were upregulated and 11 were downregulated in NEC patients. The results of the GO analysis indicated the following expression patterns: (1) biological process: the differentially expressed genes were significantly concentrated in the processes of heterologous metabolism, drug response, redox process, inflammatory reaction, and carbohydrate metabolism; (2) cell component: the differentially expressed genes were dramatically clustered in the components of membrane, exosomes, plasma membrane, components of plasma membrane, and sarcoplasmic membrane; (3) molecular function: the differentially expressed genes were concentrated in protein homodimeric activity, transport activity, actin filament binding, iron ion binding, actin binding and monooxygenase activity. Although variable factors affect the blood supply of the intestinal mucosa, ischemia-induced necrosis is still regarded as the core pathological feature in the development of NEC. In the diagnosis of NEC, nonspecific biomarkers, such as acute C-reactive protein (CRP), procalcitonin (PCT), serum amyloid A, platelet-activating factor, tumor necrosis factor-α, interleukin-6, interleukin-8 and other nonspecific biomarkers, are mediators of pro- and anti-inflammatory pathways of the immune system and play important roles in the pathogenesis of NEC. GO analysis revealed that IL-6 can be measured to distinguish NEC from nonsepticemia-related diseases, although it is difficult to distinguish NEC from septicemia. Chatziioannou et al. compared the omics data of children with NEC and children with septicemia by LC‒MS/MS mass spectrometry and identified the protein (APOA4) that could better distinguish the two diseases [14]. The results are consistent with those in this study, which suggests that APOA4 may be a gene closely related to NEC that can be employed in distinguishing NEC from some other diseases.

KEGG enrichment analysis revealed that the differentially expressed genes were significantly concentrated in metabolism, fat digestion and absorption, protein digestion and absorption, chemical carcinogenesis, carbohydrate digestion and absorption, and retinol metabolism pathways, among others. Currently, Toll-like receptor 4 (TLR4) is the most frequently studied signaling pathway in NEC. TLR4, which is highly expressed in intestinal epithelial cells of preterm mice and humans, is essential for NEC development [15, 16]. Inhibitors of TLR4 signaling include small molecules, amniotic fluid, breast milk and intestinal epithelium; the absence of TLR4 can alleviate the severity of NEC [17, 18]. TLR4, which plays a key role in the pathogenesis of NEC, is widely expressed in intestinal epithelial cells and intestinal lymphocytes [19]. After TLR4 is activated by the corresponding pathogenic microorganism, it initiates the innate immune response, furthers the downstream NF-kB signaling pathway and mediates the expression and release of inflammatory factors such as IL-1, IL-6, IL-8 and TNF-α. Studies have shown that the expression of TLR4 in the gut epithelium is increased in intestinal inflammation in human and mouse models, and overexpression of TLR4 leads to a signaling cascade that initiates nuclear translocation of NF-kB and promotes overtranscription of proinflammatory cytokines, inducing the incidence of NEC [20]. IL-6 binds with important molecules of the innate immune system to activate TLR4, which stimulates intracellular signaling and produces inflammatory cytokines. The myeloid differentiation-2 (MD-2) and GM2 activator (GM2A) proteins are members of the MD-2-related lipid recognition (ML) family. MD-2 is a very important component of the intestinal TLR4 innate immune signaling pathway [21]. MD-2 has been proven to bind with TLR4 to form a heterodimer, thus forming a complete binding site for lipopolysaccharide (LPS) [22], and hence, MD-2 is a necessary accessory molecule for TLR4 to bind with LPS [23]. Cells expressing TLR4 alone or TLR4 and mutant MD-2 showed low LPS reactivity [24]. MD-2 is an important component of the CD14-TLR4/MD-2 receptor complex, which can be used to identify the components of the bacterial cell wall [25]. Therefore, genetic polymorphisms of the MD-2 gene promoter or its exons can significantly affect the transcriptional activity of the MD-2 gene or LPS-induced signal transduction, resulting in abnormal immune responses. These findings suggested that such changes in biological processes and signaling pathways might play important roles in the evolution of NEC [26].

PPI network analysis led to the identification of key genes, the most important of which are EPCAM, CDH1, CFTR, IL-6, APOB, APOC3, APOA4, SLC2A and NR1H4. Interleukin 6 (IL6) is one of the genes encoding the cytokine interleukin family and plays roles in inflammation and B-cell maturation. In addition, it has been proven that the protein encoded by this gene mainly induces an inflammatory response by binding to interleukin-6 receptor (IL6R). It is produced at the site of acute and chronic inflammation, where it is released into serum [27]. IL6 regulates the differentiation of various cells of the immune system, including macrophages, T cells and several other cell types [28, 29]. In cells such as monocytes, macrophages, fibroblasts, and endothelial cells, IL6 expression is regulated by inflammatory pathways, such as NF-κB or activator protein 1 (AP-1). Studies by Gross et al. demonstrated that serum concentrations of IL6 in patients with inflammatory bowel disease, compared to healthy controls, increased significantly [30]. Further study found that high expression of IL6 was common in patients with Crohn's disease and ulcerative colitis and was closely associated with disease activities [31]. Louis et al. used high IL6 serum levels as biomarkers to predict recurrence cessation in patients with Crohn’s disease [32, 33]. In fact, IL6 serum levels show a higher disease activity association than the more extensively used biomarker, C-reactive protein [34]. It has been proven that IL6 expression in inflammatory bowel disease may be derived from the activation of a variety of cells, including monocytes, intestinal epithelial cells and lamina propria monocytes [35]. IL6 plays a key role in the differentiation of Th17 cells from natural CD4 + T-cell precursors and the transformation of Treg cells into IL17 + Treg cells. Ma Fei and other authors found that Treg cells producing CCR9 + IL17 in peripheral blood were significantly increased among children as well as mice with NEC. IL6 can promote the transformation of CCR9 + Treg cells back to CCR9 + IL17 + Treg cells. When the IL6 signal is blocked, transduction can inhibit this transformation [36]. CDH1 is a gene encoding the classical cadherin of the cadherin family and the protein that maintains intercellular tight connections of the intestinal tract. Current studies have focused mainly on its mutations and actions on cancer proliferation, invasion and metastasis. CDH1 has also been identified as a susceptibility gene for inflammatory bowel disease and may increase the risk of Crohn’s disease and ulcerative colitis [37, 38]. In a recent study in the Netherlands, genetic testing was conducted on 821 patients with ulcerative colitis and 1260 healthy individuals and determined that rs1728785 of CDH1 was mutated, resulting in a 1.23-fold increased risk of ulcerative colitis [39]. Similar results have been demonstrated by a study from the University of Toronto in Canada [40]. In the current research, we think that CDH1 mutation in NEC mainly affects intestinal tight junctions, as the mutation induces differentiation of the intestinal mucosal barrier. Apolipoprotein B (APOB) is a gene that encodes apolipoprotein in chylomicrons and low-density lipoprotein (LDL). It has two plasmatic subtypes, apoB-48 and apoB-100. ApoB is encoded by a single gene of a single long-chain mRNA. Recent studies are mainly limited to diseases such as those mediated by the apoB gene or its regulatory region mutation-related hypolipidemia, and few have focused on intestinal diseases.

### Limitations

Unlike using the RNA-seq technique, which sequences the whole transcriptome, the dataset used in this article only provides a profile of predefined transcripts or genes through hybridization; therefore, technically, it did not provide a full picture of gene expression. Additionally, since the dataset is not a single-cell-based array, the cell-specific gene profile is impossible to conclude (e.g., immune cells, endothelial cells, epithelial cells). In addition, the nine samples in this study constitute a very small sample size, resulting in weakened evidence.

## Conclusions

In summary, this study used bioinformatics methods to apply NEC gene chip data to the GEO database, screened the differentially expressed genes, and performed GO and KEGG enrichment analyses to identify the genes and signaling pathways that might be related to NEC. IL-6 and TLR4 may play important roles in the incidence and development of NEC. In addition, the roles of eight key genes in NEC, in addition to IL-6, are not clear, and further study is encouraged.

## Data Availability

All data generated or analyzed during this study are included in this published article and its supplementary information files.
